# Source Data for the Focus Area Maturity Model for Software Ecosystem Governance

**DOI:** 10.1016/j.dib.2020.105656

**Published:** 2020-05-12

**Authors:** Slinger Jansen, Zherui Yang

**Affiliations:** a Utrecht University, Princetonplein 5, Utrecht 3584CH, The Netherlands; b Erasmus University Rotterdam, Burgemeester Oudlaan 50, 3062 PA Rotterdam, The Netherlands

**Keywords:** Software ecosystem governance, developer ecosystems, focus area maturity models, software platform orchestration

## Abstract

We define a software ecosystem as a set of organizations collaboratively serving a market for software and services. Typically these ecosystems are underpinned by a common technology, such as an extendable software platform. This data set supports the article that describes the Software Ecosystem Governance Maturity Model ($SEG-M^{2}$) [50]. The model has the goal to support software ecosystem orchestrators in the management and governance of the actors in their ecosystems in a structured way. Through a critical structured literature review, 168 practices have been collected. These practices have been evaluated through six case studies at software ecosystem orchestrators. The practices are described with a practice code, a practice name, a practice description, required success conditions, the person responsible for the practice, and the associated literature where the practice was identified.

## 1 Specifications Table

**Table:** 

Subject	Management of Technology and Innovation
Specific subject area	A focus area maturity model for software ecosystem governance
Type of data	Text, literature references, tables
How data were acquired	Systematic literature survey and evaluation in case studies
Data format	Raw and Analyzed
Parameters for data collection	The collected practices had to fit a narrow definition of that the practice had to be executable, implementable, and understandable by a member of the platform management team.
Description of data collection	The data was collected through a literature survey that started with an SLR [46] as its source. The data was grouped according to topical similarity. Practices were subsequently evaluated by practitioners, i.e., employees at the platform orchestrators who were responsible for the success of the platform and its orchestration. If more than 2 practitioners found the practice relevant and useful, they became part of the collection. For information on selection of the practitioners, we refer to the related research article [50].
Data source location	The articles are cited in this brief. Furthermore, we report on the companies in the associated research article [50].
Data accessibility	Please find ecosystems.bib and capabilities-secoMM-2020-DiB.json attached with this article. The citation labels in the json file are matched to the ecosystems.bib file. Finally, please find an easily readible version below.
Related research article	Slinger Jansen (2019). A Focus Area Maturity Model for Software Ecosystem Governance. Information and Software Technology. https://doi.org/10.1016/j.infsof.2019.106219 (open access).

## 2 Value of the Data

• The data can be used by software ecosystem researchers for evaluation, validation, and extension of the model
• The data can be used by focus area maturity researchers to establish the vocabulary used in the field
• The data can be used by software ecosystem researchers as a basis for future research work in the domains of platform management and data ecosystem management
• The data are reusable by consultants in providing platform providers with knowledge about how to govern their ecosystem
• The data are reusable by consultants and practitioners to assess whether they have implemented a practice fully

## 3 Data

The data are a set of practices that can be used by keystone organizations to evaluate the management and governance of their ecosystems and together make up a focus area maturity model for software ecosystem governance evaluation. The practices are deeply rooted in both empirical experience, the desk studies, and literature. The practices have been described using the following elements:

• **Practice code -** The practice code is made up of three numbers. The first number concerns the focus area, the second number the capability, and the third number the maturity level. As there are empty elements in the matrix, the numbers are not consecutive.
• **Practice -** The name of the practice, as it is mentioned in the SEG-*M*^2^.
• **Focus area -** The focus area is mentioned to indicate the domain in which this practice is relevant.
• **Description -** A paragraph of text is provided to describe the practice in detail. The main reason for providing a lengthy description is internal validity: in future evaluations by third parties, they should be able to perform the evaluations independently.
• **When implemented -** Provides a series of necessary conditions before this practice can be marked as implemented. Again, to strengthen internal validity of the SEG-*M*^2^.
• **Role responsible -** One of the main findings during the case studies was that managers wanted to know who should be responsible for implementing a particular practice. This is now part of the SEG-*M*^2^ as well. The roles are indicators, as the naming in companies can be different and domain specific.
• **Literature -** Several references are given to articles that mention the practice. The literature is mainly found in the mentioned SLRs. Please note that these bibliographic entries can also be found in the data file ecosystems.bib. The citation codes used in the JSON file are referred to with their bibtex identifier.

Recently, we have created an online version of the focus area maturity model on the web site https://maturitymodels.org.

## 4 Experimental Design, Materials, and Methods

The full description of how this data was acquired is provided in the accompanying article [50]. The practices were found by taking the literature studies of Manikas [51] and Alves et al. [52] as a starting point. We analyzed the papers mentioned in these studies and identified the practices in them, by collaboratively searching through these articles and confirming the practices with both researchers. After this, we snowballed one level deeper into the existing articles and found some newer works that also contained usable practices for the maturity model.

We defined a practice as *any practice that has the express goal to change the position of the platform in the software ecosystem*, for instance by standardizing partnering capabilities. A second criterion is that the practice has to be executable by an employee of the platform orchestrator and has to have one role assigned to it as a responsible.

The SEG-*M*^2^ went through two evaluation cycles. First, the cases were evaluated against sixdesk studies, which looked at existing materials of existing companies, mostly by literature study, old case materials, and online platform descriptions. In the second cycle, the SEG-*M*^2^ was evaluated and complemented with empirical case studies, each comprising 5 days or more on site, through six software ecosystem governance maturity evaluations at four orchestrator organizations. The model was not significantly changed after the first cycle. Saturation was not purposefully reached, but the case participants indicated that the model provided an effective mechanism for the improvement of their software ecosystem management practices. Three of the case companies are still using the models to evaluate their software ecosystem management practices.

**Table:** 

Associate Models	Partner Promotion and Grooming	**Practice Code**:1.1.1	**Practice Name**:Scout strategic partners

		**Description:** Organizations must attract strategic partners who can be exemplary extenders in the ecosystem. Frequently, these have already been attracted in an earlier stage, as they may have been the ones to demand that the platform be opened in the first place.	
		**Implemented when:**• The organization has a process to continuously scout for strategic partners.	
		Literature:[1]	Responsible:Partner Manager
		**Practice Code**:1.1.2	**Practice Name**:Partner relationship model
		**Description:**Organizations must create associate models with template contracts that enable different partners to achieve partnership status.	
		**Implemented when:**• The organization has an associate model with model contracts. • The associate model has several layers for the different target groups.	
		Literature:[2], [3]	Responsible:Partner Manager
		**Practice Code**:1.1.3	**Practice Name**:Partner training and showcasing
		**Description:**Organizations can stimulate the community by regularly showing partners that other partners are successful. This can be done through regular channels, such as web sites, newsletters, partner events, and courses.	
		**Implemented when:**• The organization must have a channel to approach partners. • The organization must regularly use the channel to showcase partners.	
		Literature:[2], [3]	Responsible:Partner & Community Managers
		**Practice Code**:1.1.4	**Practice Name**:Certification
		**Description:**The organization integratively certifies partners in different categories (developers, sales, support, consultancy, training, etc.)	
		**Implemented when:**• The organization has an associate model in place. • The organization certifies partners in different categories. • Certification results are publicly available. • Partners carry and promote proof of certification.	
		Literature:[3], [4], [5]	Responsible:Partner Manager
		**Practice Code**:1.1.5	**Practice Name**:Partner health analysis
		**Description:**The organization actively monitors the health of partners and takes action when the health does not suffice. Well functioning partners are supported, sometimes even financially. Poorly functioning partners are demoted.	
		**Implemented when:**• The organization has a partner monitoring tool in place. • The organization uses the associate model to control and monitor partners.	
		Literature:[6], [7], [8], [9]	Responsible:Partner Manager
		**Practice Code**:1.1.6	**Practice Name**:Informal Consultancy Partner Support
		**Description:**The organization starts an informal consultancy partner program and outsources tasks to them. Also, the organization starts a training program and introduces consultant partner account managers.	
		**Implemented when:**• The organization provides consultants with support and training, and tightens relationships with them. • The organization monitors partner service levels at customers.	
		Literature:[10], [11]	Responsible:Partner & Community Manager
		**Practice Code**:1.1.7	**Practice Name**:Partner exclusion
		**Description:**The organization defines exclusion criteria for particular partners. Partners are excluded based on poor behavior or strategic positions in competing ecosystems.	
		**Implemented when:**• The organization has a definition for what makes a favorable partner. • The organization excludes partners that misbehave.	
Literature:[10]	Responsible: Partner Manager

**Table:** 

Associate Models	Partner Promotion and Grooming	**Practice Code**:1.2.1	**Practice Name**:Establish informal agreement with partners

		**Description:**The organization must reach an agreement with partners in terms of governing an informal partnership network. Moreover, the organization must draft procedures involved in acquiring new partners and defining the entry requirements a potential partner must meet.	
		**Implemented when:**• The organization seeks stable and legalized partnership in order to avoid unexpected conflict with partners. • The organization expects partners to perform to a certain degree and to meet the requirements.	
		Literature:[10], [11], [12]	Responsible:Partner Manager
		**Practice Code**:1.2.2	**Practice Name**:Partner contract
		**Description:**The organization must prepare sufficiently elaborated and carefully constructed contracts in order to attract high quality partners and to establish a vertical inter-firm authority relation that can subsequently guide behavior. Also, the organization must set up rules and processes to which partners must adhere and to penalize or remove partners who fail to comply.	
		**Implemented when:**• The organization selects partners for collaboration and long-term cooperation. • The organization filters and evaluates partners according to rules and regulations established in the contracts	
		Literature: [10], [11], [12]	Responsible:Partner Manager
		**Practice Code**:1.2.3	**Practice Name**:Implement an Associate Model
		**Description:**The organization implements an associate model to sustain, manage, cluster, and expand their partner ecosystem and therefore the number of actors within this ecosystem. In the meanwhile, the organization must enable coordinated collaboration and governance, requiring legal contracts and a well thought out promotion program.	
		**Implemented when:**• The organization must pro-actively design standards partnership contracts. • The organization has a partner attraction funnel in place. • The organization has partner policies in place	
		Literature: [10], [11], [12]	Responsible:Partner Manager
		**Practice Code**:1.2.6	**Practice Name**:Implement advanced associate model
		**Description:**The organization must develop partnerships with highly experienced and proven independent partners. The organization enables partners to rapidly become members through account managers. The organization minimizes effort for partners and the organization itself to be assigned as a new partner.	
		**Implemented when:**• The organization has a partner management system. • The organization automatically analyzes new partnership requests and assigns Partner Managers to new partners. • The organization uses template contracts to rapidly generate partnership agreements, and provide licenses and API keys to partners	
Literature: [10], [11], [12]	Responsible:Partner Manager

**Table:** 

Associate Models	Partner Promotion and Grooming	**Practice Code**:1.3.2	**Practice Name**:Involve Start-ups

		**Description:**The organization actively includes start-ups and tries to attract them to the ecosystem by providing new opportunities. Furthermore, the organization uses incubators, start-up funds, and universities to attract new start-ups.	
		**Implemented when:**• The organization actively attracts start-ups through start-up contests and hackathons.	
		Literature: [13], [14]	Responsible:Partner & Community Manager
		**Practice Code**:1.3.3	**Practice Name**:Consultancy Training
		**Description:**The organization must train consultants to do configuration and customization on the platform and keep track of the progress of certified engineers.	
		**Implemented when:**• The organization actively starts company academy and highly value talent. • The organization pro-actively train, certify and enable consultants to do projects with customers	
		Literature: [13]	Responsible:Partner Manager
		**Practice Code**:1.3.5	**Practice Name**:Consultant certification
		**Description:**The organization must certify third-party consultants. Their certification means that they are allowed to work on the platform at the customer.	
		**Implemented when:**• The organization establishes a complete training program for consultants and provides them with certification. • A record is kept of all certified professional	
		Literature:	Responsible:Partner Manager
		**Practice Code**:1.3.6	**Practice Name**:Organize consultant events
		**Description:**The organization must provide opportunities for consultants to exchange ideas and experiences and therefore enhances consultant professional knowledge and capabilities. The organization must organize events and meetups for consultants in order to form a healthy and positive ecosystem.	
		**Implemented when:**• The organization surrounds itself with certified consultants. • The organization values talents and enables consultants to do projects with customers. • The organization organizes events for consultants	
Literature: [3]	Responsible:Partner Manager

**Table:** 

Associate Models	Partner Promotion and Grooming	**Practice Code**:1.4.1	**Practice Name**:Direct customers to partners

		**Description:**The organization must connect and direct customers to partners according to customers’ needs and requirements. The organization helps to decide which appropriate and sufficient partners should the customers be assigned to.	
		**Implemented when:**• The organization sets up a partner management system and customer relationship management system. • The organization values partners above new customers. • The organization directs new customers to partners. • The organization informally measures partner performance with customers.	
		Literature: [2], [3]	Responsible:Partner Manager
		**Practice Code**:1.4.3	**Practice Name**:Create a partner index
		**Description:**The organization must create a partner index, such as one web site where all partners can be found in particular domains or with particular solutions. Specifically, the organization must design different domains for different partner portal pages.	
		**Implemented when:**• The organization opens up the partner management system and track partners accordingly. • The organization completes the partner profiles and improves the partner index and the system.	
		Literature: [2], [5]	Responsible:Partner Manager
		**Practice Code**:1.4.4	**Practice Name**:Provide ticketing system
		**Description:**The organization must set customer service standards with Service Level Agreements (SLAs) in ticketing system. And the organization must make sure customers always receive timely responses and all the requirement from customers will be recorded in the ticketing system with tracking and feedback. The organization also monitors the response time of partners, if they are direct contact points.	
		**Implemented when:**• The organization sets up ticketing system and track the life cycle of the tickets. • The organization responses to the customers in time and provide satisfying solutions	
		Literature: [5], [6]	Responsible:Partner Manager
		**Practice Code**:1.4.5	**Practice Name**:Provide customer contact data to partners
		**Description:**The organization must be able to access critical customer data including key contacts, communication history, and more. And the organization must have insights about how to engage with them to deals they’re involved in. Moreover, the organization must direct partners to customers by offering customer contact data.	
		**Implemented when:**• The organization actively collects customer data and connects customers to partners	
		Literature: [2], [5], [6]	Responsible:Partner Manager
		**Practice Code**:1.4.7	**Practice Name**:Share customer configurations
		**Description:**The organization must help customers to store their system configurations. The organization shares the configurations with partners and revokes their rights and knowledge when the customer switches to another partner.	
		**Implemented when:**• The organization manages the customer configurations and provides partners with configuration information	
Literature: [2], [6]	Responsible:Partner Manager & CTO

**Table:** 

Associate Models	Partner Promotion and Grooming	**Practice Code**:1.5.1	**Practice Name**:Partner and customer focus

		**Description:**The organization must focus on the need from partners and customers, and the relationship between partners and customers. Furthermore, the organization must value the need of customer and partner relationship management. The organization treats partners with the same or higher priority.	
		**Implemented when:**• The organization starts to establish partner and customer relationship management mechanism	
		Literature: [3]	Responsible:Partner Manager
		**Practice Code**:1.5.4	**Practice Name**:Co-acquisition
		**Description:**The organization collaboratively attempts to attract new customers with partners. This ensures that the customer trusts the partner and creates loyalty from the partner to the platform.	
		**Implemented when:**• The organization does collaborative sales with partners. • The organization does marketing together and shares press kits with partners	
		Literature: [5]	Responsible:Partner Manager
		**Practice Code**:1.5.5	**Practice Name**:Revenue sharing
		**Description:**The organization shares revenue with partners. When the platform is sold by a partner, the partner receives a share of that revenue, for a limited amount of time.	
		**Implemented when:**• The organization has a partner management system with a fulfillment module. • The organization has a partnership model with reseller fees and incentives	
		Literature: [3], [5]	Responsible:Partner Manager
		**Practice Code**:1.5.6	**Practice Name**:Partner focus
		**Description:**The organization is shifting its focus from its core customer group to its core partner groups. Increasingly, partners get more say in the way in which the platform and its enabling business models develop.	
		**Implemented when:**• Partners get an equal or larger say in the platform’s development than customers	
Literature: [3], [5]	Responsible:Partner Manager

**Table:** 

Associate Models	Partner Promotion and Grooming	**Practice Code**:1.6.2	**Practice Name**:Simple getting started guides

		**Description:**The organization must set up simple guidance for the start of building extensions. These guides explain how to set up the platform, how one can start developing, and how one can deliver the extension to customers.	
		**Implemented when:**• The organization creates simple guides for creating an extension. • The organization starts to form training programs	
		Literature: [10]	Responsible:Partner Manager & CTO
		**Practice Code**:1.6.3	**Practice Name**:Create a professional training organization
		**Description:**The organization must value highly of employee training and create a professional training organizations, such as company academy. Also, the organization must set requirement for employees to get training for promotion. In addition, the organization must train specific types of staff, including domain specialists, technical or business consultants and sales partners.	
		**Implemented when:**• The organization values talents and intents to cultivate talents within the organization for the purpose of loyalty. • The organization starts establishing company academy and certifying developers and consultants. • The organization also certifies organizations based on the amount of training their employees have had	
		Literature: [3], [5]	Responsible:Partner Manager
		**Practice Code**:1.6.4	**Practice Name**:Certification based on training
		**Description:**The organization must approve the outcome of professional employee training program. And therefore, the organization must provide either internal or external certification based on training.	
		**Implemented when:**• The organization improves the training program by adding certified approval. • The organization values talents and thinks of employees’ future career path	
		Literature: [10]	Responsible:Partner Manager
		**Practice Code**:1.6.6	**Practice Name**:Partner employee management
		**Description:**The organization maintains a record of all certified professionals working at partners in the ecosystem. Their data is kept up to date, so that when particular knowledge is needed in a region, the platform provider can supply potential candidates.	
		**Implemented when:**• A record is kept of all certified professionals, including their employers	
Literature: [3], [5]	Responsible:Partner Manager

**Table:** 

Associate Models	Partner Promotion and Grooming	**Practice Code**:1.7.1	**Practice Name**:Informal sales partner support

		**Description:**The organization actively prepares sales packages for partners. Furthermore, the organization does collaborative sales meetings, helps partners to stimulate start-ups and selects preferred consultancy partners.	
		**Implemented when:**• The organization prepares sales support packages. • The organization tries to improve sales success rates for partners	
		Literature: [3], [5]	Responsible:Partner Manager
		**Practice Code**:1.7.3	**Practice Name**:Certify sales partners
		**Description:**The organization must provide partners with sales support, send leads to partners and start sales training. Partner employees that are trained are certified.	
		**Implemented when:**• The organization has a formal incentive scheme and partnership program. • The organization certifies sales partners.	
		Literature: [3], [5]	Responsible:Partner Manager
		**Practice Code**:1.7.4	**Practice Name**:Create market-specific sales groups
		**Description:**The organization must assign particular sales experts and others to specific domains. In the meanwhile, the organization must collect and share data about domains to create market-specific sales groups.	
		**Implemented when:**• The organization sorts specialists according to different domains and realizes the significance of market-specific sales	
		Literature: [15], [16]	Responsible:Partner Manager
		**Practice Code**:1.7.5	**Practice Name**:Organize local sales events
		**Description:**The organization must organize local sales events, such as sales groups workshop. Moreover, the organization must gather sales to participant sales events for communication and idea-exchange.	
		**Implemented when:**• The organization builds complete sales organism to attract high-quality partners	
		Literature: [15], [16]	Responsible:Partner & community Manager
		**Practice Code**:1.7.7	**Practice Name**:Partner awards
		**Description:**The organization must value the performance of partners and thus must award partners who have performed well in the collaboration and cooperation. In the meanwhile, the organization must rate partners according to their performance and filter out those who have performed poorly. The partner awards are a wonderful opportunity for international publicity on the achievements of partners.	
		**Implemented when:**• The organization establishes standard to evaluate partners performance and rates them in order to sort out valuable and less desirable partners. • The organization organizes partner award events	
Literature: [3], [5]	Responsible:Partner Manager

**Table:** 

Associate Models	Partner Promotion and Grooming	**Practice Code**:2.1.1	**Practice Name**:Informal tests extensions for partners

		**Description:**The organization tests extensions informally for partners. Also the organization supports developers in creating their own tests and must provide partners with exemplary testing methods.	
		**Implemented when:**• The organization provides partners with testing methods and evaluate testing tools.	
		Literature: [3], [5], [9]	Responsible:Partner & Quality manager
		**Practice Code**:2.1.2	**Practice Name**:Create extension/app test procedure
		**Description:**The organization must provide extension developers with procedures and tools for extension testing, and typical test scenarios. Moreover, the organization must ask developers to submit their test cases for extension certification.	
		**Implemented when:**• The organization provides partners with established testing practices and has a discussion about requesting test scripts for certification.	
		Literature: [3], [9], [17], [18]	Responsible:Quality Manager
		**Practice Code**:2.1.4	**Practice Name**:Binary application test procedure
		**Description:**The extensions delivered by extenders are tested in a binary manner, i.e., the source code is not checked. These binary checks can range from simple (has the extension been updated and have only a couple of bytes been changed?) to extensive black box testing.	
		**Implemented when:**• A test environment is built that pre-tests extensions before they can be released to customers.	
		Literature: [3], [17], [18]	Responsible:Quality Manager
		**Practice Code**:2.1.5	**Practice Name**:Allow extenders to self-test
		**Description:**The organization must provide partners with guidance and procedures to perform self-test. Moreover, the organization must offer assistance when partners run into problems or barriers when doing self-test.	
		**Implemented when:**• The organization has well structured guidance for self-test. • The organization has back-end support for partners to solve problems	
		Literature: [3], [5]	Responsible:Quality manager
		**Practice Code**:2.1.7	**Practice Name**:Partners submit tests with App
		**Description:**Partners deliver the extension to the organization with tests, to show that they have sufficient coverage and that the extension is well tested.	
		**Implemented when:**• The organization has a system that can receive and automate test scripts. • The organization has an infrastructure to test extensions	
Literature: [3], [5]	Responsible:Quality manager

**Table:** 

Associate Models	Partner Promotion and Grooming	**Practice Code**:2.2.1	**Practice Name**:Support partners with quality

		**Description:**The organization must help partners to guarantee the application quality and support partners to avoid or solve potential quality issues. Moreover, the organization must form special groups or support teams.	
		**Implemented when:**• The organization provides quality guidelines to partners	
		Literature: [3], [5]	Responsible:Quality manager
		**Practice Code**:2.2.3	**Practice Name**:Platform sandbox
		**Description:**The organization must establish an environmental platform sandbox that developers can use to simulate the features and characteristics of the production environment. Moreover, the organization must create simulated responses from the applications relying on the platform and test the applications’ reaction.	
		**Implemented when:**• The organization relies on the platform as the core in a way as a database or user interface are considered core components to the architecture. • The organization wants to fully test the performance of the platform	
		Literature: [20], [21]	Responsible:CTO & Product Manager
		**Practice Code**:2.2.4	**Practice Name**:Detect quality issues
		**Description:**The organization must identify quality issues in extensions and the platform and report these back to the extension developers.	
		**Implemented when:**• The organization test drives extensions and reports quality issues back to the developers.	
		Literature: [19], [20], [21]	Responsible:Quality & Community Managers
		**Practice Code**:2.2.5	**Practice Name**:Share issues with partners
		**Description:**The organization must share issues detected in the platform. In addition, the organization must give partners visibility into the work stream and restrict visibility of issues within a project.	
		**Implemented when:**• The organization targets to fully engage partners in the development process. • The organization collaborates with partners closely and aims to form a healthy and trustworthy relationship	
		Literature: [3], [5]	Responsible:Quality & Community Managers
		**Practice Code**:2.2.7	**Practice Name**:Create operation knowledge portals
		**Description:**The organization provides extension developers with knowledge of how the extension performs in the field.	
		**Implemented when:**• The organization provides partners with dashboards and reports about how the extension performs in the field. • Error and crash reports are sent to the extension developer	
Literature: [22]	Responsible:Quality Manager

**Table:** 

Associate Models	Partner Promotion and Grooming	**Practice Code**:2.3.1	**Practice Name**:Informal contacts

		**Description:**The organization maintains informal contacts with developers and can contact them directly if needed. The partner and the platform provider collaborate closely to deliver the platform and its extensions.	
		**Implemented when:**• The organization maintains informal contacts with developers	
		Literature: [2], [3], [5], [23]	Responsible:Community & Partner Managers
		**Practice Code**:2.3.2	**Practice Name**:Developer meet-ups are organized
		**Description:**The organization creates opportunities for developers to exchange information, for instance by organizing developer meetups.	
		**Implemented when:**• The organization organizes events for developers and after the discussion or idea exchange, developers provide insight and feedback for innovation	
		Literature: [2], [3], [24]	Responsible:Community & Partner Manager
		**Practice Code**:2.3.3	**Practice Name**:Feedback channels are coordinated
		**Description:**The organization must establish ways and methods for feedback from developers in order to help with decision-making and influences product roadmap. Moreover, the organization must utilize the feedback to provide developers with the requirements they want.	
		**Implemented when:**• The organization measures developers satisfaction and coordinates the channels for developers to provide feedback	
		Literature: [2], [3], [5]	Responsible:Community & Product Managers
		**Practice Code**:2.3.4	**Practice Name**:Developer interaction is supported
		**Description:**The organization creates opportunities for developers to exchange information, discusses new releases and features of the platform, and presents innovation technology road map for the organization. Developers interact about the latest features and provide insight into their usage scenarios.	
		**Implemented when:**• There are forums, besides meetings, where developers can interact	
		Literature: [2], [3], [23]	Responsible:Community & Quality managers
		**Practice Code**:2.3.6	**Practice Name**:Partners help partners
		**Description:**The organization must establish a mechanism for partners to seek help from other partners. In other words, the organization must form a community or channel a method for partners to exchange knowledge and to offer help freely.	
		**Implemented when:**• The organization has long-term cooperation and collaboration with partners. • The organization provides a channel, such as an on-line forum, for partners to support each other	
		Literature: [3], [5], [25], [26]	Responsible:Community & Partner Managers
		**Practice Code**:2.3.7	**Practice Name**:Developers can contribute to other developers
		**Description:**The organization creates opportunities for developers to contribute to each other’s software, for instance by providing development tools and supportive libraries. The organization must allow developers to interact with each other and to contribute within the community.	
		**Implemented when:**• The organization encourages developers to develop or add components to the platform.	
Literature: [2], [3], [26], [27]	Responsible:CTO & Community Manager

**Table:** 

Associate Models	Partner Promotion and Grooming	**Practice Code**:2.4.2	**Practice Name**:Quick install for SDK

		**Description:**The organization must provide easy download, install and upgrades for SDK. The organization optimizes the ‘time to first hello world’ actively, ideally under 15 minutes.	
		**Implemented when:**• The organization monitors the process and decreases the number of steps	
		Literature: [18], [28]	Responsible:Release Manager
		**Practice Code**:2.4.4	**Practice Name**:Automated testing
		**Description:**The organization must automate repetitive but necessary tasks in a formalized testing process, or perform additional testing that would be difficult to do manually. Test automation is critical for continuous delivery and continuous testing.	
		**Implemented when:**• The organization uses separate software to control the execution of platform tests	
		Literature: [18], [27], [28]	Responsible:Test & Release Managers
		**Practice Code**:2.4.5	**Practice Name**:IDE extensions
		**Description:**The organization delivers IDE extensions to make development easier. These extensions can range from simple SDKs, tool tips, and even complete new IDEs to integrate all the features from a particular platform.	
		**Implemented when:**• The organization delivers support tools for the IDE that partners use	
		Literature: [9], [18], [22], [28]	Responsible:Release Managers
		**Practice Code**:2.4.6	**Practice Name**:Automated releasing
		**Description:**The organization must combine the capabilities of deployment automation, environment management and modeling, and release coordination. Moreover, the organization must package, deploy, and update an application from development, across various environment, and to production.	
		**Implemented when:**• The organization helps to provide a combination of automation, environment modeling and work-flow management capabilities. • The organization helps to deliver software rapidly, reliably and responsibly	
Literature: [18], [22], [28]	Responsible:Release manager & CTO

**Table:** 

Associate Models	Partner Promotion and Grooming	**Practice Code**:2.5.1	**Practice Name**:Informal development partner support

		**Description:**The organization provides extension developers with informal support, mostly through uncoordinated communication channels.	
		**Implemented when:**• The organization has informal contacts to extension developers	
		Literature: [3], [5]	Responsible:Partner Manager
		**Practice Code**:2.5.2	**Practice Name**:Dedicated engineers
		**Description:**There are dedicated partner engineers who support extension developers with their problems. These partner engineers collaborate with partners and occasionally visit the partner.	
		**Implemented when:**• The organization hires developers that support the partners only	
		Literature: [2], [3], [22]	Responsible:CTO
		**Practice Code**:2.5.3	**Practice Name**:Knowledge infrastructure for partners
		**Description:**The organization must provide partners with knowledge database for frequently asked questions, ticket system, developer community or forum, requirements infrastructure, and road maps. Also, the organization must allow partners to rate whether the content is useful or not.	
		**Implemented when:**• The organization actively seeks feedback from partners and provide relevant information for partners. • The organization pro-actively establishes knowledge infrastructure for partners in order to get better feedback and consequently build better cooperation with partners.	
		Literature: [3], [5]	Responsible:Partner Manager & CTO
		**Practice Code**:2.5.4	**Practice Name**:Ticketing systems
		**Description:**The organization must take incoming requests for support and automatically generates a service ticket. Also, the organization must provide with a consistent ticket, making ticket management much easier to quickly solve the issue to their satisfaction.	
		**Implemented when:**• The organization wants to provide with consistent service. • The organization wants to track all relevant data over time, allowing support teams to learn and improve the support they provide	
		Literature: [2], [3]	Responsible:CTO & Quality Manager
		**Practice Code**:2.5.5	**Practice Name**:Collaborative road mapping
		**Description:**The organization performs collaborative road mapping and delegates features to partners. When feature conflicts arise, the organization contacts the partner and attempts to find a solution.	
		**Implemented when:**• The organization enables cross-functional teams to collaborate in the roadmapping process. • The organization shares planning information with suppliers and partners to support open innovation.	
		Literature: [3], [5], [13], [29]	Responsible: Partner Manager & CTO
		**Practice Code**:2.5.6	**Practice Name**:Collaborative development
		**Description:**The organization must adapt a collaborative development model to identify possibilities for collaborations with partners. If possible, the platform developer delegates work to the partners.	
		**Implemented when:**• The organization delegates features to extension developers and partner	
		Literature: [13], [14]	Responsible:Community Manager & CTO
		**Practice Code**:2.5.7	**Practice Name**:Facilitate ecosystem of ecosystems
		**Description:**The organizations enables partners to create their own ecosystems, such as the Games Workshops of the Steam platform or the GreaseMonkey script ecosystem around Firefox.	
		**Implemented when:**• The platform enables third parties to create their own ecosystems around their extension. • The organization legally allows new ecosystems to bloom around its technology	
Literature: [3]	Responsible:Community Manager & CTO

**Table:** 

Associate Models	Partner Promotion and Grooming	**Practice Code**:2.6.1	**Practice Name**:Informal transparency

		**Description:**The organization provides informal transparency into the requirements for the platform, and its road map.	
		**Implemented when:**• Partners receive notice about new requirements and platform plans.	
		Literature: [2], [3], [5]	Responsible:Community & Partner Managers
		**Practice Code**:2.6.2	**Practice Name**:Formal communications policy for requirements
		**Description:**The organization must direct all traffic for requirements and make formal communications policy for requirements.	
		**Implemented when:**• The organization implements a requirements management tool and uses its communication channels to communicate requirements with developers	
		Literature: [9], [27], [30]	Responsible:Release & Product Managers
		**Practice Code**:2.6.3	**Practice Name**:Provide Requirements Infrastructure
		**Description:**The organization must provide partners with access rights to the insight of requirements management according to roles. Moreover, the organization must share requirements with appropriate partners and collect partner’s high-priority requirements.	
		**Implemented when:**• The organization helps to guide partners in requirement participation.	
		Literature: [18], [22], [28]	Responsible:Release Manager & CTO
		**Practice Code**:2.6.4	**Practice Name**:Partner plays part in requirements portal
		**Description:**The organization must make requirements database open to partners, in order to allow partners to participate in requirements engineering. Furthermore, the organization must inform partners with their roles in requirement portal.	
		**Implemented when:**• The organization gives partners a voice in prioritization and in requirements. • The organization provides partners with insight into requirements rejection and acceptance	
		Literature: [3], [5], [31]	Responsible:Release & Product Managers
		**Practice Code**:2.6.5	**Practice Name**:Partners support prioritization
		**Description:**Partners provide their input in the prioritization of features on the road map. Partners provide feedback and expect feedback on their feedback.	
		**Implemented when:**• Partners provide feedback on the road map. • Tools are made available that enable a partner to vote up or down features	
		Literature: [3], [5], [9]	Responsible:Release & Product Managers
		**Practice Code**:2.6.7	**Practice Name**:Partners pick up requirements as co-developers
		**Description:**The organization must initiate and support partners to pick up requirements as co-developers using strategies that developed an infrastructure for continuous improvement and inquiry. Moreover, the organization must support and nurture co-developers’ capacities individually and as a community of learners to help them work effectively.	
		**Implemented when:**• The organization sees partners as co-developers	
Literature: [3], [5], [13], [14], [31]	Responsible:Product Manager & CTO

**Table:** 

Associate Models	Partner Promotion and Grooming	**Practice Code**:2.7.2	**Practice Name**:Open road map of the platform for developers

		**Description:**The organization must provide partners with insight into the short term road map for the platform.	
		**Implemented when:**• The road map is shared with extension developers	
		Literature: [27], [29], [30]	Responsible:Community, Release & Product Managers
		**Practice Code**:2.7.5	**Practice Name**:Partner extensions taken into account
		**Description:**Increasingly, the organization behind the platform is taking into account partner extensions when creating the road map. When deciding to cut major features from a platform, the key partners using the feature are contacted.	
		**Implemented when:**• Road map creating includes an inventory of the types of extensions that are disabled and enabled by changes in the feature set. • Extension developers are contacted before major changes to the platform. • Every potential new feature is first evaluated within the ecosystem: has this already been built by one of our main partners	
		Literature: [3], [5], [29]	Responsible:Product & Community Managers
		**Practice Code**:2.7.6	**Practice Name**:Partner extensions part of strategic road map
		**Description:**The organization collaborates with partners and includes their requirements and road maps on the platform road map. In this way, road maps can be synchronized.	
		**Implemented when:**• The organization creates a combined road map to offer competitive features collaboratively with partners.	
Literature: [3], [5], [32]	Responsible:Partner & Release Managers & CTO

**Table:** 

Associate Models	Partner Promotion and Grooming	**Practice Code**:2.8.1	**Practice Name**:Informal monitoring of developers

		**Description:**The organization monitors developers, observing their behavior, either in an IDE or by organizing informal sessions for developers.	
		**Implemented when:**• The organization must ask developers about their goals and needs	
		Literature: [15], [16]	Responsible:Quality Manager & CTO
		**Practice Code**:2.8.2	**Practice Name**:Monitor developers and their motives
		**Description:**The organization monitors developers for their motives and to keep up to date on market developments. The organization focuses on finding out about the usage scenarios, to make sure that the platform adds value for the partners.	
		**Implemented when:**• The organization organizes dev-sessions and hackathons. • The organization talks to developers and their bosses on site	
		Literature: [15], [16]	Responsible:Community & Product Managers
		**Practice Code**:2.8.4	**Practice Name**:Document feedback from developers
		**Description:**The organization must pay attention to the need from developers by checking question on platforms, such StackOverflow and developer fora, talking to developers and performing surveys. Moreover, the organization must ask developers to write developer blogs and document what developers need and wants in order to track their motives and need.	
		**Implemented when:**• The organization constantly checks on developers and monitors their journey towards their achievements. • The organization constantly ask developers to blog about their experiences and performs developer surveys	
		Literature: [15], [16]	Responsible:Quality, Community,& Product Managers
		**Practice Code**:2.8.5	**Practice Name**:Adjust documentation according to demands
		**Description:**The organization must keep track on developers’ knowledge requirements and adjust documentation accordingly once demands change.	
		**Implemented when:**• The organization collects developer requirements and adjusts documentation accordingly	
		Literature: [22], [33]	Responsible:Quality Manager
		**Practice Code**:2.8.6	**Practice Name**:Study developer behavior through SOK
		**Description:**Developer behavior is watched closely by monitoring developer behavior in different settings. Data is collected on how developers build software extensions, where they experience problems, and what mistakes they make. The data is typically collected automatically, although it is also common that development methods are evaluated at Hackathons and developer meetings.	
		**Implemented when:**• The organization collects software operation knowledge. • The organization collects software operation knowledge on developer behavior. • The organization analyzes and acts upon this knowledge by improving manuals and code.	
		Literature: [18], [28]	Responsible:Quality & Product managers
		**Practice Code**:2.8.7	**Practice Name**:Use automatic data collection from IDE
		**Description:**Data is collected on the developer’s behavior through the IDE. This is especially easy when the IDE is cloud based and hosted by the platform provider.	
		**Implemented when:**• The organization collects software operation knowledge from the IDE used by its extenders	
Literature: [18], [28]	Responsible:Quality & Product managers

**Table:** 

Associate Models	Partner Promotion and Grooming	**Practice Code**:3.1.1	**Practice Name**:Internal extensions list

		**Description:**The organization maintains an informal list of extensions created by extenders. The list is informal and is usually updated by hand.	
		**Implemented when:**• An informal extension list is kept.	
		Literature: [18], [27], [28]	Responsible:Community & Partner Managers
		**Practice Code**:3.1.3	**Practice Name**:List of extensions
		**Description:**The organization maintains a list of extensions in the run up to an app store or app delivery platform and publishes the list to outsiders. In the mean time, the organization must consider quality rating from customers and develop mechanism for approving extensions into the list.	
		**Implemented when:**• The organization creates a list of extension with links to partners and uses it for demo purposes to win over extenders	
		Literature: [18], [27], [28]	Responsible:Community & Partner Manager
		**Practice Code**:3.1.5	**Practice Name**:App Store
		**Description:**The organization creates marketplaces for applications that are available for download and purchase. These are presented through a market mechanism, such as an app store.	
		**Implemented when:**• The organization allows developers to sell and distribute their products to actors within one or more multi-sided software platform ecosystems	
		Literature: [17]	Responsible:Release & Product Managers & CTO
		**Practice Code**:3.1.6	**Practice Name**:Microservice architecture
		**Description:**The organization designs software applications as suites of small independently deployable services, each running in its own process and communicating with lightweight mechanisms, to enable scalable architectures. Also, the organization must build these services around business capabilities.	
		**Implemented when:**• The organization builds applications as suites of services. • Third party services are adopted in the ecosystem through an orchestration framework	
		Literature:[34]	Responsible:Chief Architect
		**Practice Code**:3.1.7	**Practice Name**:Dynamic app composition
		**Description:**The organization must define an application as being dependent on another application, such as middleware or a plug-in. Also, the organization includes mechanisms to orchestrate the interaction among applications and therefore provides functionality to program the behavior of the active space. When all mechanisms are in place, apps can self-select dependent extensions, to dynamically create new solutions	
		**Implemented when:**• The organization provides an architecture with pre-defined interfaces that enables automated app composition. • Based on customer problems, new compositions are created by an intelligent extension or automated service composer	
Literature: [28], [35]	Responsible:Chief Architect & Product Managers & CTO

**Table:** 

Associate Models	Partner Promotion and Grooming	**Practice Code**:3.2.2	**Practice Name**:Integrative components, manual installation

		**Description:**Components and extensions are installed, but manually. Considerable work is involved in integrating the extension, sometimes requiring glue code to make it work.	
		**Implemented when:**• Extensions can easily be integrated, but require manual work	
		Literature: [15], [16]	Responsible:Chief Architect & Release & Product Managers & CTO
		**Practice Code**:3.2.4	**Practice Name**:One-click install of integration
		**Description:**Extensions can be installed without complicated installation procedures. Extensions are typically made available through an app store or App Index. The delivery mechanism has been perfected to manage the extension as a separately managed component to the platform.	
		**Implemented when:**• Extensions can be installed with one click, similar to apps	
		Literature: [18], [28]	Responsible:Chief Architect & Release & Product Managers & CTO
		**Practice Code**:3.2.5	**Practice Name**:On-demand applications
		**Description:**Software extensions can be installed without interference of a partner or the platform owner. Customers install the applications when they need them and can delete them independently.	
		**Implemented when:**• Applications can be installed automatically, for instance using a dependency mechanism	
		Literature: [3], [17], [18], [18], [28]	Responsible:Chief Architect & Release & Product Managers & CTO
		**Practice Code**:3.2.6	**Practice Name**:Extendable applications
		**Description:**The organization allows applications that themselves can be adapted by third parties. Furthermore, the platform enables new extension architectures to be developed on top of it.	
		**Implemented when:**• The platform enables the extension of existing applications	
Literature: [3], [17], [18]	Responsible:Chief Architect & Release & Product Managers & CTO

**Table:** 

Associate Models	Partner Promotion and Grooming	**Practice Code**:3.3.2	**Practice Name**:Informal approval process of extensions

		**Description:**Extensions are informally approved through an internal approval procedure.	
		**Implemented when:**• There exists a series of guidelines on what to do when a new extension is delivered	
		Literature: [3], [5]	Responsible:Quality & Product Managers & CTO
		**Practice Code**:3.3.4	**Practice Name**:Establish app approval team
		**Description:**The organization must form an application approval team to review applications and extensions.	
		**Implemented when:**• There is an app approval team that approves extensions. • If rejected, the team discusses with extenders how the extension can be improved	
		Literature: [3], [17], [18]	Responsible:Quality & Product Managers & CTO
		**Practice Code**:3.3.5	**Practice Name**:Process support and automation
		**Description:**The organization must orchestrate and integrate tools, people and processes through work flow and make automated processes for app submission and testing.	
		**Implemented when:**• The organization automates the extension approval process, to reduce human error and achieve scale	
		Literature: [3], [17], [18]	Responsible:Quality & Product Managers & CTO
		**Practice Code**:3.3.6	**Practice Name**:Self-regulation through app appraisal by end-users
		**Description:**The organization must allow end-users to rank and comment on the applications they provide and adjust the product based on the feedback and all the appraisal from end-users.	
		**Implemented when:**• The organization values the feedback from end-users and practice self-regulation on application development.	
		Literature: [3], [17], [18]	Responsible:Quality & Product Managers & CTO
		**Practice Code**:3.3.7	**Practice Name**:App approval process with external partners
		**Description:**The organization involves third parties, such as National health services, to approve apps and extensions, by recognizing these types of parties as valuable members of the ecosystem.	
		**Implemented when:**• The organization attracts third parties to support extension approval	
Literature: [5]	Responsible:Quality & Product Managers & CTO

**Table:** 

Associate Models	Partner Promotion and Grooming	**Practice Code**:3.4.3	**Practice Name**:Opportunistic

		**Description:**Extensions are opportunistically approved or rejected. There exists no formal approval policy yet.	
		**Implemented when:**• The organization evaluates new extensions pragmatically	
		Literature: [27], [30]	Responsible:Quality & Product Managers & CTO
		**Practice Code**:3.4.4	**Practice Name**:Set formal rules
		**Description:**The organization must establish formal rules, which are most often found in end user license agreements (EULAs) that prohibit particular behaviors such as reverse engineering and copyright infringement. Also, the organization must also include benchmarks in a particular manner.	
		**Implemented when:**• The organization sets regulations to protect and prevent particular detrimental behaviors of partners	
		Literature: [27], [30]	Responsible:Quality & Product Managers & CTO
		**Practice Code**:3.4.6	**Practice Name**:Appeals policy
		**Description:**Extenders can appeal extension rejections. Appeals are studied closely to find problems with the appeal and a discussion is started with the extension developer about how the extension can be made acceptable.	
		**Implemented when:**• There exists a formal appeals policy that partners can use to recall decisions from the orchestrator.	
		Literature: [27], [30]	Responsible:Quality & Product Managers & CTO
		**Practice Code**:3.4.7	**Practice Name**:Community curation support
		**Description:**The community curates apps and provides feedback on them. When the community aggressively complains about the poor quality of an extension, the platform supplier takes action.	
		**Implemented when:**• The organization monitors community ratings of apps. • The organization takes appropriate action when too many complaints are filed	
Literature: [15], [16]	Responsible:Quality & Community Managers & CTO

**Table:** 

Associate Models	Partner Promotion and Grooming	**Practice Code**:3.5.5	**Practice Name**:Marketing of extensions in app store

		**Description:**The organization must allow applications to market their apps in top 10 lists and ‘recommended’ apps categories.	
		**Implemented when:**• The organization allows extensions to be marketed in the extension market.	
		Literature: [15], [16]	Responsible:Product Manager & CTO
		**Practice Code**:3.5.6	**Practice Name**:Marketing of extensions outside of app store
		**Description:**Extensions and apps are advertised through other channels than through the app store, such as tv commercials and internet advertising.	
		**Implemented when:**• The organization promotes apps outside of the scope of the platform	
Literature: [15], [16]	Responsible:Product & Partner Managers

**Table:** 

Associate Models	Partner Promotion and Grooming	**Practice Code**:3.6.3	**Practice Name**:Create developer forum

		**Description:**The organization creates a forum for developers to post questions and share comments with fellow developers and application engineers. Such a forum can be independent, or on a web site such as StackOverflow or the Chinese Developer Network (CDN). Moreover, the organization must provide developers with a variety of development topics, from getting started to working with the latest beta software.	
		**Implemented when:**• The organization creates a support community through a developer forum	
		Literature: [2], [3], [3], [5]	Responsible:Community manager & CTO
		**Practice Code**:3.6.5	**Practice Name**:Organize development conferences and hackathons
		**Description:**The organization must identify the need from developers and organize conferences, meetups, and hackathons for developers.	
		**Implemented when:**• The organization encourages developers to share and learn from their peers. • The organization identifies possible contributions among developers. • The organization regularly organizes events in new domains, that combine learning opportunities with development	
		Literature: [2], [3], [3], [5]	Responsible:Community & Partner Managers & CTO
		**Practice Code**:3.6.6	**Practice Name**:Showcase developers and solutions
		**Description:**The organization regularly showcases developers and their solutions through different channels. Typically, developers are invited to come present their solutions at conferences and hackathons.	
		**Implemented when:**• Developers are invited to showcase their solutions through different channels	
		Literature: [27], [30]	Responsible:Community & Partner Managers
		**Practice Code**:3.6.7	**Practice Name**:Showcase libraries and SDKs from developers
		**Description:**The organization must create showcase inventories for developers, including benchmarks and specific SDKs, in order to enrich applications with advanced functionalities, advertisements, push notifications and more. The showcase libraries could include SDKs to interface to a particular programming language or to include sophisticated hardware.	
		**Implemented when:**• The organization demonstrates and offers software libraries from partner developers to other developers	
Literature: [27], [30]	Responsible:Community & Partner Managers & CTO

**Table:** 

Associate Models	Partner Promotion and Grooming	**Practice Code**:3.7.2	**Practice Name**:Implement a Reseller Model

		**Description:**The organization can resell apps of others. Also, third parties can resell services and apps of others. The organization sends a newsletter to partners, promoting apps implemented at competitors.	
		**Implemented when:**• The organization creates incentive schemes, as to mobilize partners to do sales for the platform owner as well.	
		Literature: [15], [16]	Responsible:Partner Manager & CTO
		**Practice Code**:3.7.4	**Practice Name**:app store model
		**Description:**The organization offers a curated market that allows extenders to monetize their applications and software.	
		**Implemented when:**• The organization implements an extension market	
		Literature: [17]	Responsible:Product Manager & CTO
		**Practice Code**:3.7.5	**Practice Name**:In-app purchases
		**Description:**The organization enables provision of content and special subscriptions that end-users can buy in applications. The purchasing process is completed directly from within the app and is seamless to the user in most cases.	
		**Implemented when:**• The organization provides novel business models and in-app purchasing	
		Literature: [17]	Responsible:Product Manager & CTO
		**Practice Code**:3.7.7	**Practice Name**:Subscription
		**Description:**The organization offers extenders to create subscriptions. End-users can subscribe to the extra services accordingly.	
		**Implemented when:**• Extenders are able to create subscriptions related to their extension.	
Literature: [17], [27]	Responsible:Product Manager & CTO

**Table:** 

Associate Models	Partner Promotion and Grooming	**Practice Code**:4.1.1	**Practice Name**:Local products licensed

		**Description:**The organization must own the intellectual property of products before authorizing to manufacture them. The licensed product may be supplied under its original name, or a different one.	
		**Implemented when:**• The organization legally protects its own intellectual property.	
		Literature: [36], [37]	Responsible:Product & Release Managers
		**Practice Code**:4.1.6	**Practice Name**:Sharing licenses with partners
		**Description:**The organization provides partners with access to licenses for the product manufacturing and publishing. Also, the organization must share the intellectual property of some products.	
		**Implemented when:**• The organization agrees with partners for higher transparency over intellectual property and collaboration	
		Literature: [5], [36], [37]	Responsible:Product & Partner Managers
		**Practice Code**:4.1.7	**Practice Name**:Automated checking of license violations
		**Description:**The organization checks license violations. In addition, the organization must prioritize the intellectual property share and violation in order to avoid legal problems.	
		**Implemented when:**• The organization owns several license and needs automated checking to keep license valid. • All submitted extensions are checked for license violations, based on the extensions currently available in the organization’s market	
Literature: [25], [36]	Responsible:Product & Release Managers

**Table:** 

Associate Models	Partner Promotion and Grooming	**Practice Code**:4.2.1	**Practice Name**:Reuse policy for internal products

		**Description:**The organization has a policy for the usage of code, existing software, or software knowledge, in order to save time and resources and reduce redundancy by taking advantage of assets that have already been created in some form within the software product development process.	
		**Implemented when:**• The organization follows principles of reusability. • The organization uses the part of the written programs in the construction of other programs	
		Literature: [16]	Responsible:CTO
		**Practice Code**:4.2.2	**Practice Name**:Reuse policy for external products
		**Description:**There is a reuse policy within the organization about which products from closed and open source can and cannot be reused. The policy is shared with all developers, so it is always clear what components are part of the framework and which intellectual property is owned by third parties.	
		**Implemented when:**• The organization has a reuse policy that states which kinds of artifacts can and cannot be reused within the platform	
		Literature: [16], [27], [30], [37], [38]	Responsible:CTO & Partner Manager
		**Practice Code**:4.2.3	**Practice Name**:Reuse policy for internal products with partners
		**Description:**The organization establishes a reuse policy for all artifacts that can be used by extenders and partners. Some characteristics that make software more easily reusable are modularity, loose coupling, high cohesion, information hiding and separation of concerns.	
		**Implemented when:**• The organization enables extenders to reuse artifacts for the platform	
		Literature: [5], [16]	Responsible:CTO & Partner Manager
		**Practice Code**:4.2.4	**Practice Name**:Reuse policy for external products with partners
		**Description:**The reuse policy that is shared within the company is also shared with partners. In this way, partners are aware of which components to use in their extensions without violating intellectual property deals within the ecosystem.	
		**Implemented when:**• There exists an extensive reuse policy that is shared with partners and extenders	
		Literature: [5]	Responsible:CTO & Partner Manager
		**Practice Code**:4.2.7	**Practice Name**:Contributions to other ecosystems coordinated
		**Description:**The organization analyzes contributions to other ecosystems and coordinates these contributions. It explicitly decides which platforms can be contributed to and which platforms are off limits for reasons of competition.	
		**Implemented when:**• The organization provides consistent contribution guidelines to other relevant software ecosystems.	
Literature: [25], [38], [39], [40]	Responsible:CTO & Partner Manager

**Table:** 

Associate Models	Partner Promotion and Grooming	**Practice Code**:4.3.4	**Practice Name**:Third party patents licensed

		**Description:**The organization must draft license agreements with third parties for commercializing patent rights, which could be the source of substantial income for the organization. Moreover, the organization allows partners to be aware of the license agreement with the third party.	
		**Implemented when:**• The organization licenses third party innovations and uses them in the platform	
		Literature: [36]	Responsible:Community & Product Managers & CTO
		**Practice Code**:4.3.5	**Practice Name**:Patents created for the platform
		**Description:**The organization must support patent related processes and creates new intellectual property for the platform. The patents are used to protect the platform and as an indication to outsiders that the platform is continuously innovated on.	
		**Implemented when:**• Patents are created for technologies within the platform	
		Literature: [15], [16]	Responsible:CTO
		**Practice Code**:4.3.6	**Practice Name**:IP sharing with partners
		**Description:**Partners get access to source code, patents, etc. These are provided to partners to enable them to innovate beyond the scope of the platform.	
		**Implemented when:**• The organization shares intellectual property with partners. • The organization distributes innovations that it does not use to its partners	
		Literature: [5]	Responsible:CTO
		**Practice Code**:4.3.7	**Practice Name**:Patent violations identified
		**Description:**The organization must prohibit the act of patent infringement with respect to a patented invention or product. Therefore, the organization must be able to identify possible violations for patent use according to local jurisdiction.	
		**Implemented when:**• The organization identifies patent infringement and coordinates violations when they occur	
Literature: [27], [36]	Responsible:Quality Manager & CTO

**Table:** 

Associate Models	Partner Promotion and Grooming	**Practice Code**:5.1.2	**Practice Name**:First weaknesses identified in architecture

		**Description:**The organization must evaluate the architecture and starts identifying weaknesses in its platform architecture. As extenders typically have less experience developing with the platform, they need to be instructed and provided a foolproof architecture.	
		**Implemented when:**• The organization researches tickets and errors; examining of developer mistakes	
		Literature: [22], [27], [38]	Responsible:Chief Architect & CTO
		**Practice Code**:5.1.3	**Practice Name**:Guards built in
		**Description:**The organization implements guard mechanisms, such as throttles, to the platform. Furthermore, notifications mechanisms are used to alert the platform developer of any misuse or security breaches.	
		**Implemented when:**• Guard mechanisms are implemented, such as throttles and security monitors	
		Literature: [19], [20]	Responsible:Chief Architect & CTO
		**Practice Code**:5.1.4	**Practice Name**:Structural hardening process
		**Description:**The organization must harden the platforms that are visible to the public in order to minimize the risk of successful attacks against it. Moreover, the organization must continue this hardening process to improve the structure of the platform and the systems.	
		**Implemented when:**• The organization has a process in place for regular penetration testing, security evaluations, and architecture improvements	
		Literature: [41]	Responsible:Chief Architect & CTO
		**Practice Code**:5.1.5	**Practice Name**:Architecture becomes first class citizen
		**Description:**The organization must evaluate and improve the robustness and security of the platform architecture.	
		**Implemented when:**• The organization puts the highest priority on creating an extendable, durable, transparent, robust architecture.	
Literature:[19], [20]	Responsible: Chief Architect & CTO

**Table:** 

Associate Models	Partner Promotion and Grooming	**Practice Code**:5.2.1	**Practice Name**:SDK or API

		**Description:**The organization must allow developers to use a software development kit (SDK) or application program interfaces (APIs) for extensions to communicate with the platform.	
		**Implemented when:**• The organization enables platform extensions through an SDK or API.	
		Literature: [18], [28], [42]	Responsible:Chief Architect & CTO
		**Practice Code**:5.2.2	**Practice Name**:Multi-layered extension framework
		**Description:**The organization introduces a multi-layered extensible security framework that enables extension of different functional domains of the platform.	
		**Implemented when:**• The organization creates different entry points into the features of the platform, dependent on the extension scenarios it imagines	
		Literature: [21]	Responsible:Chief Architect & CTO
		**Practice Code**:5.2.4	**Practice Name**:IDE Support
		**Description:**The organization reduces setup time to increase developers productivity and lower barriers to entry.	
		**Implemented when:**• The organization maximizes developers productivity by providing IDE support, such as providing an IDE plug-in dedicated to the platform	
		Literature: [18], [28]	Responsible:Chief Architect & CTO & Product Manager
		**Practice Code**:5.2.7	**Practice Name**:Fourth party extensions
		**Description:**Extenders are allowed to build their own sub-ecosystems within the ecosystem. Examples include Steam, where game developers for the Steam platform can ask fourth parties to build extensions and content for their games. The ecosystem is in these cases further stretched to partners of partners, who can receive another share of the revenue.	
		**Implemented when:**• Extenders are allowed to build their own sub-ecosystems within the ecosystem	
Literature: [2], [3], [3], [5]	Responsible:Chief Architect & CTO & Product Manager

**Table:** 

Associate Models	Partner Promotion and Grooming	**Practice Code**:5.3.2	**Practice Name**:SOK gathering about platform use

		**Description:**The organization identifies the most frequently-used software using user tracking, error messages, downloads, calls, and reported bugs. The organization must then publish prioritized lists and act upon this.	
		**Implemented when:**• The organization gathers Software Operation Knowledge about how the platform is used by end users. • The organization identifies the most common uses of the platform and simplifies the knowledge for extenders	
		Literature: [42]	Responsible:Product & Quality Managers
		**Practice Code**:5.3.4	**Practice Name**:SOK gathered about App performance
		**Description:**The organization gathers operation knowledge about extensions as well. The knowledge is communicated back to the extenders.	
		**Implemented when:**• The organization gains knowledge about the performance, quality and usage of software in the field at end-users, as well as the users’ opinions on the software	
		Literature: [42]	Responsible:Product & Quality Managers
		**Practice Code**:5.3.5	**Practice Name**:Sharing bugs and crashes
		**Description:**The organization collects crash information about extensions and shares these with the extension developer.	
		**Implemented when:**• The organization gathers software operation knowledge and crashes and shares the crash information and potential bugs with extension developers	
		Literature: [43]	Responsible:Product & Quality Managers & CTO
		**Practice Code**:5.3.6	**Practice Name**:Sharing usage
		**Description:**The organization collects software operation knowledge and reports back to extenders on this knowledge.	
		**Implemented when:**• The organization provides extension usage reports to extension developers	
		Literature: [5], [43]	Responsible: Product & Quality Managers & CTO
		**Practice Code**:5.3.7	**Practice Name**:Sharing customer configurations
		**Description:**The organization shares customer configuration knowledge with partners. In this way, organizations can service customers of large platforms. SAP, for instance, can provide configuration information to a new service provider within several hours.	
		**Implemented when:**• The organization collects customer configuration knowledge. • The organization manages which partner gets access to each customer configuration	
Literature: [43]	Responsible:Product & Partner Managers & CTO

**Table:** 

Associate Models	Partner Promotion and Grooming	**Practice Code**:5.4.1	**Practice Name**:Documentation with getting started

		**Description:**The organization must present the preliminary design of the platform documentation. Also the organization replicates and share the document at a wide scale.	
		**Implemented when:**• The organization provides documentation that helps developers start first development	
		Literature: [33]	Responsible:Product Manager & CTO
		**Practice Code**:5.4.2	**Practice Name**:Documentation with examples
		**Description:**The organization makes examples available in the documentation. Examples should illustrate common patterns using sample scenarios. Moreover, each sample scenario is inspired by a realistic extension. This document briefly describes each example and provides a link to the source code.	
		**Implemented when:**• The organization shares examples and uses code samples in the documentation	
		Literature: [33]	Responsible:Product Manager & CTO
		**Practice Code**:5.4.3	**Practice Name**:Documentation generated from code
		**Description:**The document and code are developed in parallel. Hereby, the documentation can be generated from the code. In this way documentation is easier to maintain, as code and manuals evolve in lockstep.	
		**Implemented when:**• The organization uses a system that automatically generates documentation from particular tags in the code.	
		Literature: [33]	Responsible:Product Manager & CTO
		**Practice Code**:5.4.4	**Practice Name**:Interactive documentation
		**Description:**The documentation must become interactive, in that the code samples can change language when someone indicates that they are using a particular platform.	
		**Implemented when:**• The documentation is offered in an interactive manner, where it adjusts when the extender selects a particular technology	
		Literature: [33]	Responsible:Product Manager & CTO
		**Practice Code**:5.4.5	**Practice Name**:Prioritization based on knowledge needs
		**Description:**The organization must facilitate to prioritize the documentation based on whether the content included in the document is needed the most. Also, the organization must sort out the knowledge and categorize them according to the prioritization.	
		**Implemented when:**• Documentation is shared and ordered based on popularity. • Documentation is ordered based on software operation knowledge	
		Literature: [33]	Responsible:Product Manager
		**Practice Code**:5.4.6	**Practice Name**:Feedback gathered
		**Description:**The organization gathers feedback on the quality of documentation.	
		**Implemented when:**• The organization gathers feedback on the quality of documentation	
Literature: [33]	Responsible:Quality & Community Manager & CTO

**Table:** 

Associate Models	Partner Promotion and Grooming	**Practice Code**:5.5.2	**Practice Name**:App Security Scans

		**Description:**The organization must provide all parties in the ecosystem with instructions on how to enforce platform and extension security, offer specialized security scans, and provide developers with a security newsletter. The organization involves all parties in security activity and makes it a collaboration effort.	
		**Implemented when:**• The organization provides security instructions and documentation for app developers. • The organization regularly inform partners of security issues, tips and tricks	
		Literature: [19], [20], [21], [44]	Responsible:Quality manager & Chief Architect
		**Practice Code**:5.5.3	**Practice Name**:Create Security Procedures
		**Description:**The organization must create security procedures to all parties in the ecosystem, including for partners who deploy systems.	
		**Implemented when:**• The organization actively instructs app developers and operators for security. • Documentation is provided on how to enforce security	
		Literature: [27], [30], [44]	Responsible:Quality manager & Chief Architect
		**Practice Code**:5.5.5	**Practice Name**:Security policies shared with partners
		**Description:**The organization must share security policies as well as current threats with partners and manage incidents on a secure platform. Moreover, the organization must allow partners and participants to share information in real time as the simulation developed, identify emerging cyber-attacks and weaknesses, and respond accordingly.	
		**Implemented when:**• The organization has a distributed, inter-operable information systems for partners to share security challenges	
		Literature: [5], [44]	Responsible:Partner & Community Managers
		**Practice Code**:5.5.6	**Practice Name**:Security certification of partner components
		**Description:**The organization provides security certification of extensions. By offering security certification, extenders can learn how to make their extensions safer for the platform.	
		**Implemented when:**• The organization certifies third party components on security	
		Literature: [3], [5]	Responsible:Chief Architect & CTO & Partner Manager
		**Practice Code**:5.5.7	**Practice Name**:Security alerts shared throughout ecosystem
		**Description:**The organization has a mechanism that responds to immediate detection of compromises and alerts others inside the ecosystem of the situation. It shares discoveries in an information exchange format that can be authenticated.	
		**Implemented when:**• The organization forwards the alert directly into the ecosystem if the security issue comes from external entity. • Security alerts affecting the platform are announced to all platform developers.	
Literature: [19], [20], [21]	Responsible:Chief Architect & CTO

**Table:** 

Associate Models	Partner Promotion and Grooming	**Practice Code**:5.6.4	**Practice Name**:Establish evolution policy

		**Description:**The organization has an evolution policy for the platform. In order to better identify evolution process and impact, the organization should have clear awareness and understanding of each phase of the evolution process of the platform. Moreover, the organization must draw well-established evolution policies to help manage and govern the platform for further development and improvement. By doing so, extension developers are not constantly bombarded with new features, while innovative features still make it into the platform. An example of such a policy is maintaining backwards compatibility at all times.	
		**Implemented when:**• The organization establishes clear criteria to define each process of evolution or to understand each phase of evolution progress. • The organization constitutes evolution policies for platform evolution evaluation and managerial governance.	
		Literature: [45], [46], [47]	Responsible:Chief Architect & CTO & Release Manager
		**Practice Code**:5.6.7	**Practice Name**:Direct feedback to extenders about platform versions
		**Description:**The organization sets up a mechanism to provide extenders with information about the evolution of the platform.	
		**Implemented when:**• The organization monitors the usage and operation of different versions of the platform.	
Literature:[17]	Responsible: Chief Architect & CTO & Release Manager

**Table:** 

Associate Models	Partner Promotion and Grooming	**Practice Code**:6.1.1	**Practice Name**:Informal competition analysis

		**Description:**The organization must look at the industry as a whole, compare competitors and perform individual evaluations. Furthermore, the organization examines competing platforms and copies valuable features to make sure no other platform becomes unique in its features	
		**Implemented when:**• The organization performs informal competitive analysis.	
		Literature: [48]	Responsible:Partner & Community Managers
		**Practice Code**:6.1.3	**Practice Name**:Reference Competitors Developed
		**Description:**The organization must figure out the strengths and market position of reference competitors. In the meanwhile, the organization must monitor, follow and surpass reference competitors.	
		**Implemented when:**• The organization creates a list of reference competitors and watches them closely. • The organization follows reference competitors example and copies good ideas.	
		Literature: [27]	Responsible:Community & Partner Managers
		**Practice Code**:6.1.5	**Practice Name**:Policy for contributing to other ecosystems
		**Description:**The organization must decide the policy whether or not to open up the governance. The strategic level of policy is to deal with competition and other ecosystems. Moreover, the organization must decide to contribute or even integrate into other ecosystems. Furthermore, the organization must create a knowledge management strategy to enable and empower partners in software ecosystem.	
		**Implemented when:**• The organization has an explicit policy about contributing to other ecosystems	
		Literature: [24], [27], [30], [45]	Responsible:Community & Partner Managers & CTO
		**Practice Code**:6.1.6	**Practice Name**:Domain engineering and niche discovery
		**Description:**The organization must constantly scout organizations to find high potential partners that can provide benefit to the platform. Furthermore, the organization must uncover opportunities in application domains of the platform, to see whether new opportunities can be found for partners. In this way sub-domains for the ecosystem can be found where new partners can connect to the platform and add value to it.	
		**Implemented when:**• The organization is looking for relevant domains where its platform can be applied. • The organization collaborates with partners to develop solutions within new domains	
		Literature: [15], [16]	Responsible:CTO & Product Manager
		**Practice Code**:6.1.7	**Practice Name**:Partners guided in contributions to other ecosystems
		**Description:**The organization must provide tips and tricks for partners on how to contribute to related ecosystems. The organization can stimulate partners to contribute, for instance, to open source to other ecosystems. This is different from 6.1.5, as that concerns only the internal organization and not the partners.	
		**Implemented when:**• The organization guides partners for direct contribution for specific purposes	
Literature: [5], [38], [45]	Responsible:CTO & Partner Manager

**Table:** 

Associate Models	Partner Promotion and Grooming	**Practice Code**:6.2.1	**Practice Name**:Market analysis for platform

		**Description:**The organization must evaluate the market constantly to see which domains are getting less or more popular, such as health became for Apple’s iPhone recently.	
		**Implemented when:**• The organization provides in-depth analysis of market trends, macro-economic indicators and governing factors along with market attractiveness.	
		Literature:[15], [16]	Responsible:Community & Partner Managers
		**Practice Code**:6.2.3	**Practice Name**:Share Market Data with Partners
		**Description:**The organization must share with partners relevant information about the market and convince partners of the health of ecosystem. Moreover, the organization must elaborate on the number of customers in their ecosystem and perform lead generation with partners.	
		**Implemented when:**• The organization actively provides market information and informs partners about opportunities. • The organization collaboratively performs business development with partners.	
		Literature:[3], [5], [16]	Responsible:Community & Partner Managers
		**Practice Code**:6.2.5	**Practice Name**:Customer surveys
		**Description:**Customer feedback is collected by the organization about the platform and its surrounding extensions.	
		**Implemented when:**• Customer feedback is collected by the organization.	
		Literature: [15], [16]	Responsible:Community Manager
		**Practice Code**:6.2.6	**Practice Name**:Automated data collection
		**Description:**The organization uses software operation knowledge to establish the health of the ecosystem.	
		**Implemented when:**• The organization automatically obtains software operation knowledge.	
		Literature:[22], [27], [33]	Responsible:Quality, Community & Partner Managers
		**Practice Code**:6.2.7	**Practice Name**:Customer data shared
		**Description:**The organization must enables diverse partners to develop strategy through the sharing of data. The organization establishes information partnerships.	
		**Implemented when:**• The organization uses customer data to inform extenders of potential new customers.	
Literature:[15], [16]	Responsible:Quality, Community & Partner Managers

**Table:** 

Associate Models	Partner Promotion and Grooming	**Practice Code**:6.3.2	**Practice Name**:Ask partners for performance data

		**Description:**The organization must do structural analysis of how partners are performing economically, what are the motives, and business cases. This is done to to see how much value they are adding to the ecosystem.	
		**Implemented when:**• The organization analyzes the performance data from partners.	
		Literature: [5]	Responsible:Partner Manager
		**Practice Code**:6.3.3	**Practice Name**:Strategic Partner Analysis
		**Description:**The organization must identify and promote strategic partners, assigning them to core domain specific groups. Furthermore, the organization must have partners provide input for the platform, using them for release validation and do co-development with partners. If necessary, the organization must potentially demote unsuccessful partners.	
		**Implemented when:**• The organization creates partner indexes. • The organization identifies strong partners, giving them access and status, and weak partners, demoting unsatisfactory ones	
		Literature: [5]	Responsible:Community & Partner Managers
		**Practice Code**:6.3.6	**Practice Name**:Partner surveys
		**Description:**The organization performs partner surveys. It translates questions into actionable outcomes for the business by collecting surveys from partners to get business insights. Also, the organization must use survey as a tool for building partner engagement and encouraging partners to be part of the process. Partners remain in the ecosystem if they know their opinions are valued as an essential aspect to the process.	
		**Implemented when:**• The organization performs partner surveys to establish the health of the ecosystem.	
Literature: [5], [9]	Responsible:Community & Partner Managers

**Table:** 

Associate Models	Partner Promotion and Grooming	**Practice Code**:7.1.2	**Practice Name**:Standard adoption

		**Description:**The organization adopts domain specific standards and enables developers to integrate through these standards, such as XML, REST, JSON, etc. The organization must minimize developer’s astonishment during development, and standards can be of use here.	
		**Implemented when:**• The organization implements and promotes standards developers expect	
		Literature: [27], [30]	Responsible:Chief Architect & CTO
		**Practice Code**:7.1.4	**Practice Name**:Participation in standards bodies
		**Description:**The organization actively and strategically takes part in standards bodies, and supports and funds them in the creation of modern new standards. The organization asserts its innovativeness and intellectual capital by contributing to open standards and consortia. Open Innovation	
		**Implemented when:**• The organization has an open innovation strategy. • The organization participates strategically in standards bodies.	
		Literature:[27], [30]	Responsible:Chief Architect & CTO
		**Practice Code**:7.1.7	**Practice Name**:Creation of new standards
		**Description:**The organization must create new standards to gain a strategic advantage over competitors.	
		**Implemented when:**• The organization creates new standards.	
Literature: [30], [48]	Responsible:Chief Architect & CTO

**Table:** 

Associate Models	Partner Promotion and Grooming	**Practice Code**:7.2.3	**Practice Name**:Academic Contacts

		**Description:**The organization must establish contacts with local academic institutions and start university collaboration and acquire research funding.	
		**Implemented when:**• The organization collaborates with universities. • The organization attracts research funding	
		Literature: [13], [30]	Responsible:Chief Architect & CTO & Community Manager
		**Practice Code**:7.2.5	**Practice Name**:Collaboration in research projects
		**Description:**The organization partakes in advances research projects and consortia. This enables the organization to stay innovative.	
		**Implemented when:**• The organization takes part in academic and industrial innovation collaborations.	
		Literature: [5], [13], [30]	Responsible:Chief Architect & CTO & Community Manager
		**Practice Code**:7.2.7	**Practice Name**:Shared research and development center
		**Description:**The organization creates an innovation center where partners, the organization, and startups can collaborate. The organization invests in R&D to create new and innovative products and add features to old products and services.	
		**Implemented when:**• The organization has a dedicated RnD facility. • The organization attracts third parties to work in the RnD facility.	
Literature: [5], [13], [30]	Responsible:Chief Architect & CTO & Community Manager

**Table:** 

Associate Models	Partner Promotion and Grooming	**Practice Code**:7.3.2	**Practice Name**:Stimulate in-company innovation

		**Description:**The organization gathers ideas about the platform and identify those who are internally developing solutions. Moreover, the organization organizes contests for local staff to build smart solutions and get them to develop against the platform.	
		**Implemented when:**• The organization has the community manager reach out to local users of the platform and tighten relationships with them. • The organization organize feedback sessions internally and local hackathons	
		Literature: [30]	Responsible:Community Manager
		**Practice Code**:7.3.3	**Practice Name**:Promotion of partner solutions to other developers
		**Description:**The organization promotes extensions and tools to other extenders, for instance to show the success of other partners. Moreover, the organization provides customer case examples to partners and extenders. In particular tools that speed up development are shared often and early.	
		**Implemented when:**• The organization actively looks for the best developer stories and the most innovative solutions. •The organization actively promotes solutions and encourages partners to sell software to partners	
		Literature: [5], [9]	Responsible:Community & Partner Managers
		**Practice Code**:7.3.4	**Practice Name**:Show partner innovations to partners
		**Description:**The organization must foster a culture of innovation. Therefore, the organization must show cutting-edge or valuable innovations of partners as examples to partners and cultivate the atmosphere of coming up with new ideas and worthwhile breakthroughs.	
		**Implemented when:**• The organization sets innovation display cases for partners to lure and attract more innovations from and for the ecosystem.	
		Literature: [5], [49]	Responsible:Community & Partner Managers
		**Practice Code**:7.3.6	**Practice Name**:Reward new innovations
		**Description:**The organization stimulates new innovations within the ecosystem and rewards new ideas and thoughts to bring about a positive loop of innovation and development. Moreover, the organization evaluates innovations carefully. The innovation reward compensates part of the risk of failure.	
		**Implemented when:**• The organization values innovations and formalizes a mechanism to reward innovation.	
Literature: [49]	Responsible:Community Managers & CTO

**Table:** 

Associate Models	Partner Promotion and Grooming	**Practice Code**:7.4.2	**Practice Name**:Informal sharing of technology road maps

		**Description:**The organization shares with developers the mission and the vision of the company, including the most important technical innovations over the next years. The organization must explain the technologies developers can expect in order to excite, engage and lure them.	
		**Implemented when:**• The organization organizes developers events to get feedback from them on the road maps. • The organization adds information to newsletters to show developers what they can expect from the platform.	
		Literature: [5], [30]	Responsible:Product & Release Managers
		**Practice Code**:7.4.4	**Practice Name**:Formal road map presentation
		**Description:**The organization organizes conferences or meetings to present the road map. Also the organization discusses ideas and road maps with partners and participants in the ecosystem. Moreover, the organization is open to all valuable innovations and improvement suggestions from others. Finally, the organization makes sure the road map items are published when they need to be published and avoids information leaks.	
		**Implemented when:**• The organization organizes and presents road maps in a formal way. • The organization has a communication policy regarding the road map.	
		Literature: [32]	Responsible:Product & Release Managers
		**Practice Code**:7.4.7	**Practice Name**:Collaborative road maps
		**Description:**The organization shares road maps with partners and makes their tools and solutions part of the road map. In this way, customers are aware of the partner solution co-evolution.	
		**Implemented when:**• The organization creates road maps in collaboration with strategic partners. • Partner road maps are taken into account and collaboratively coordinated.	
Literature: [5], [9], [32]	Responsible:Product & Release Managers

## 5 Declaration of Competing Interest

None.

## Appendix A Supplementary materials

Supplementary Data S1 Supplementary Raw Research Data. This is open data under the CC BY license http://creativecommons.org/licenses/by/4.0/ Supplementary Data S1
